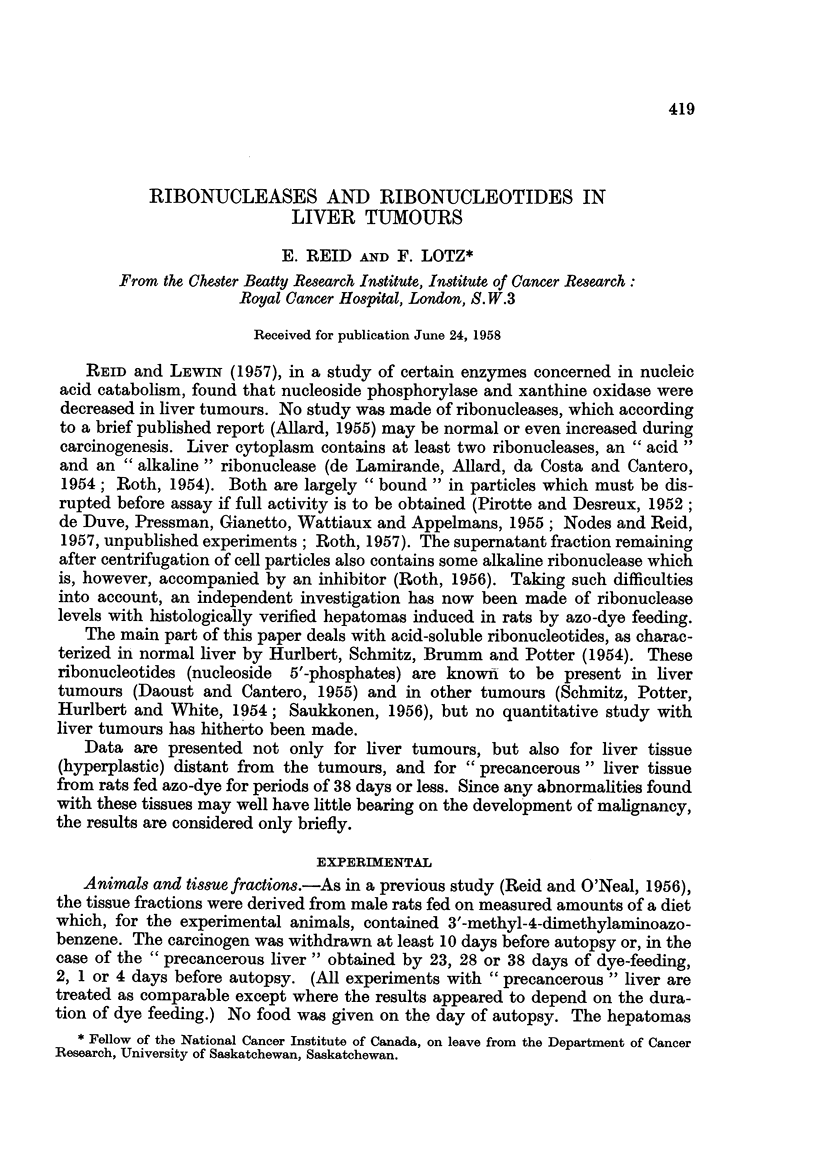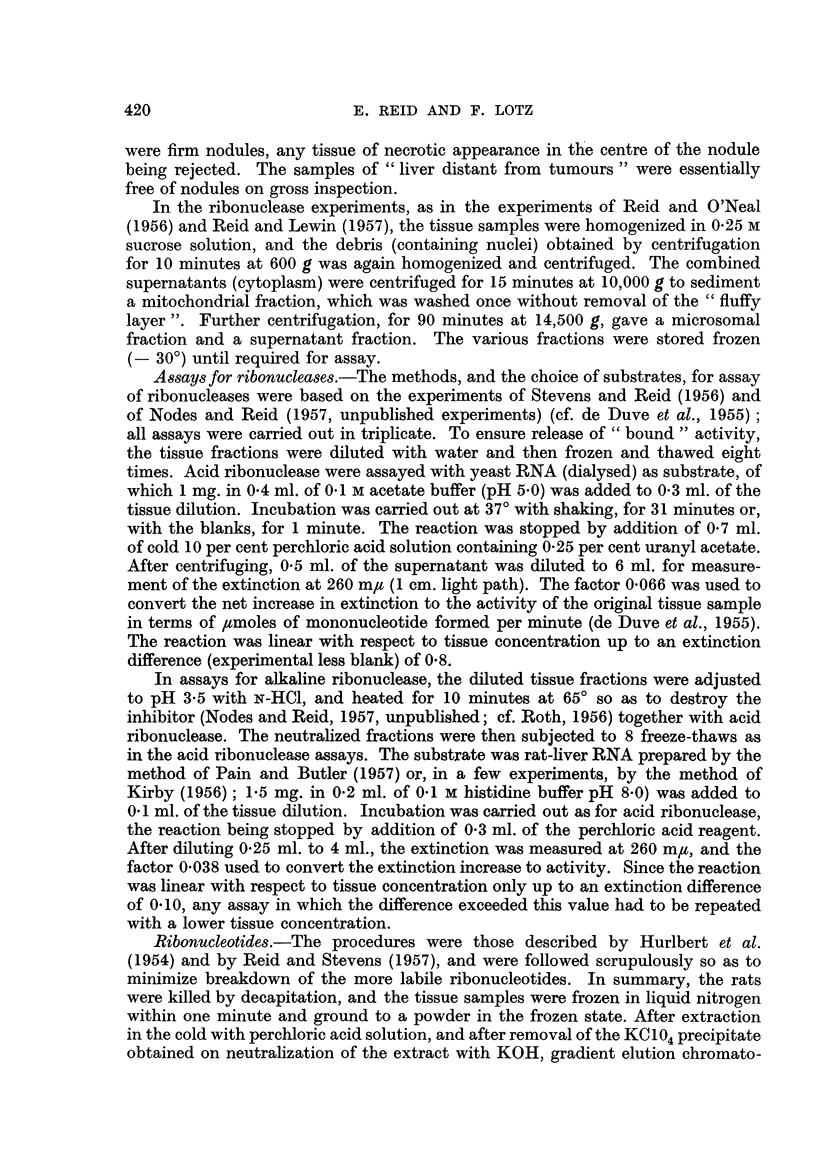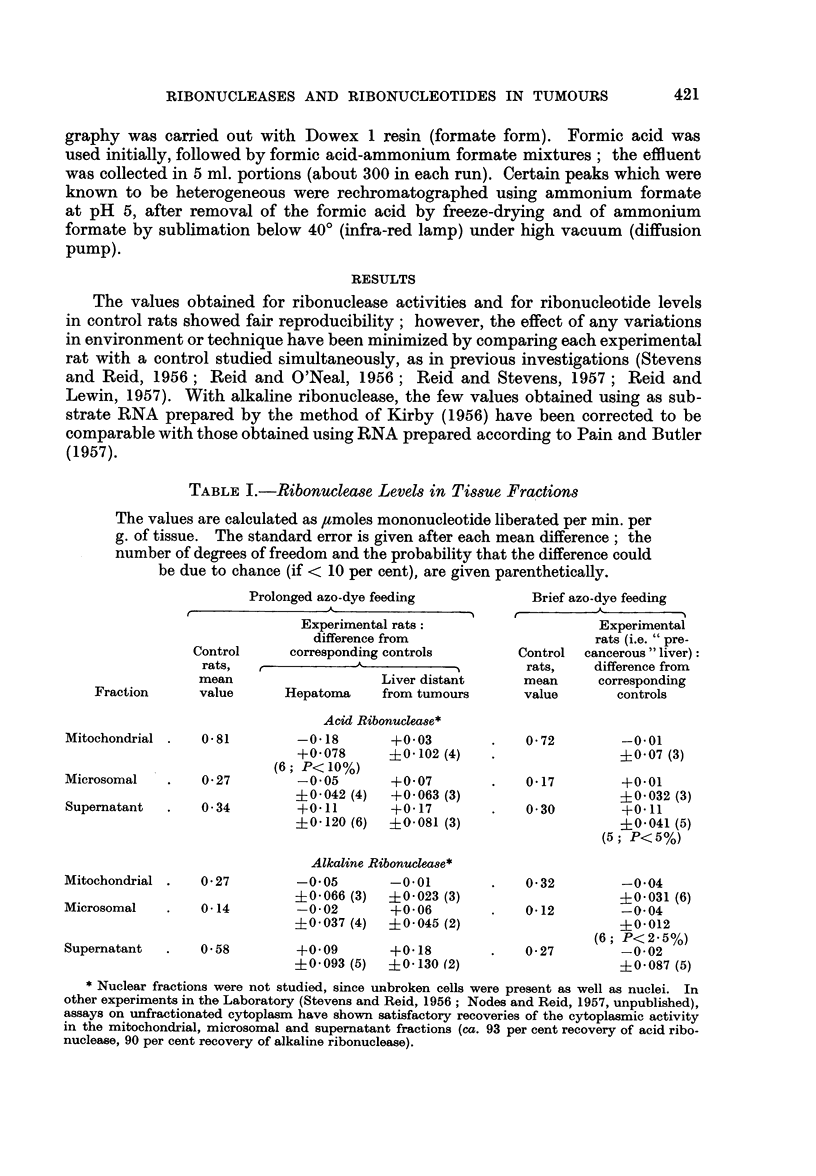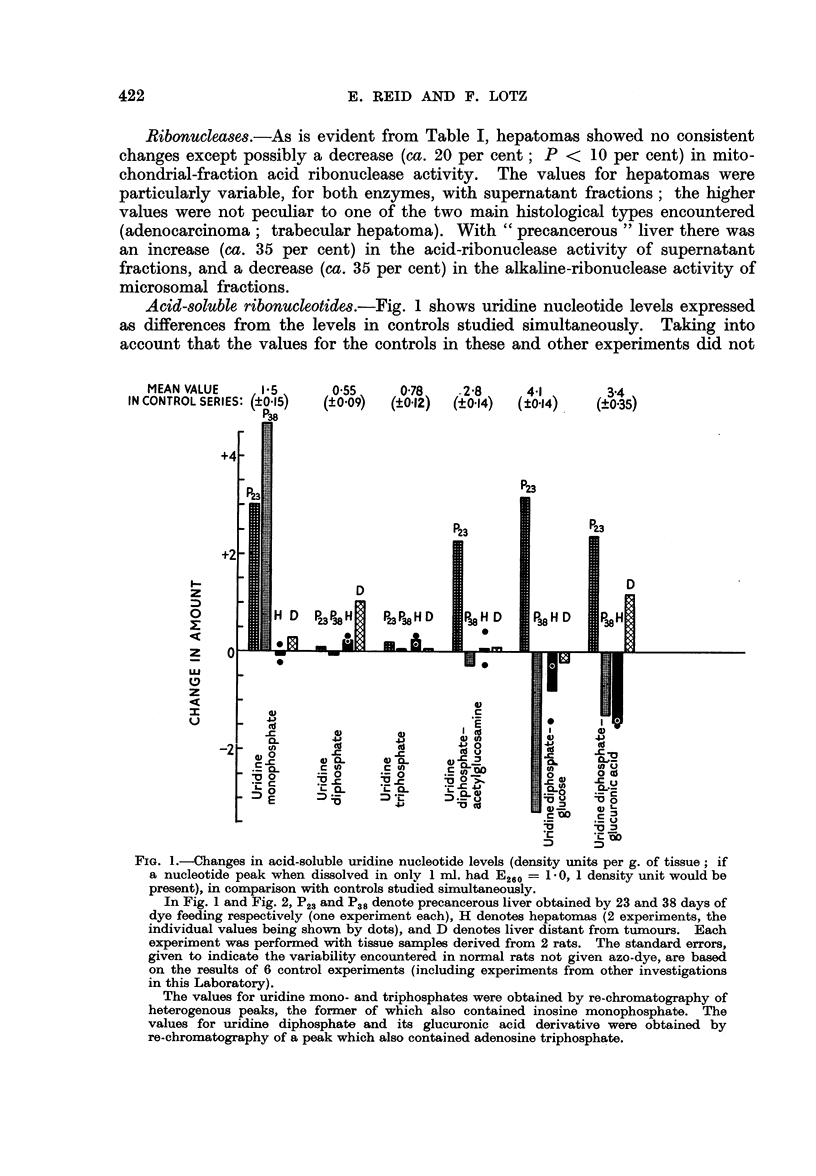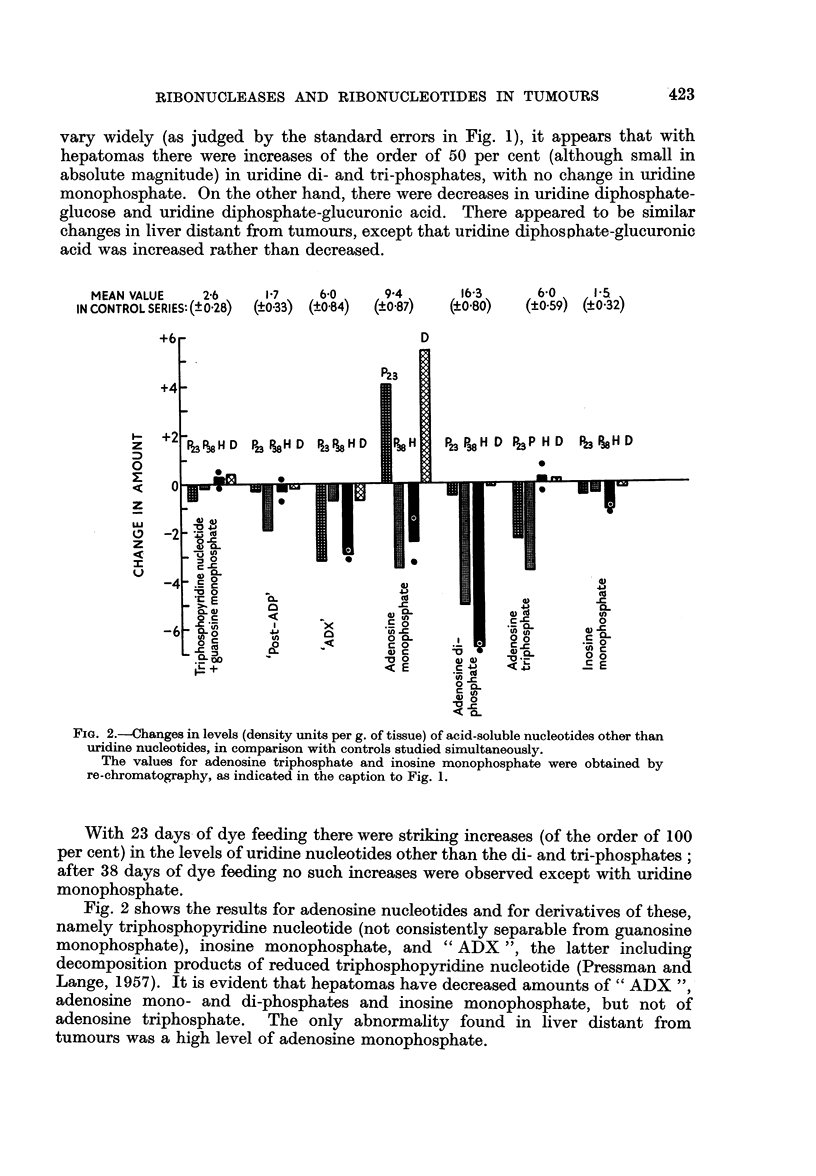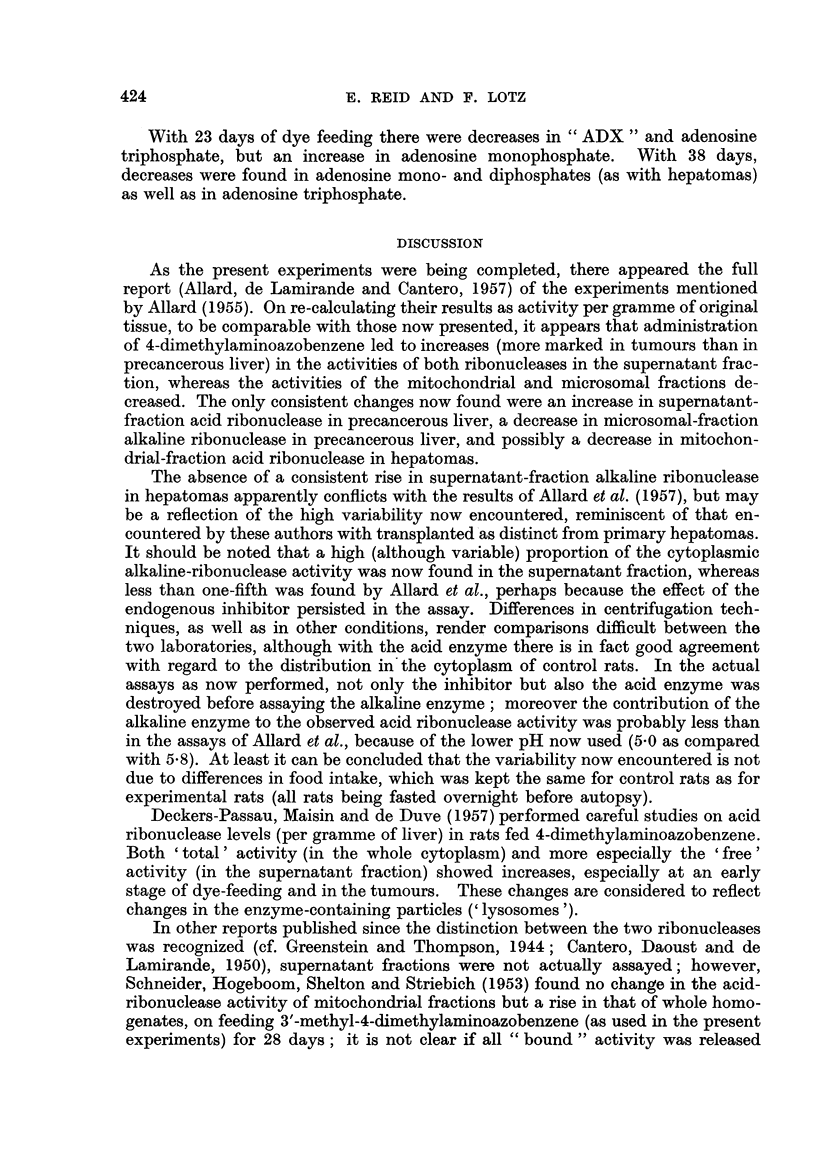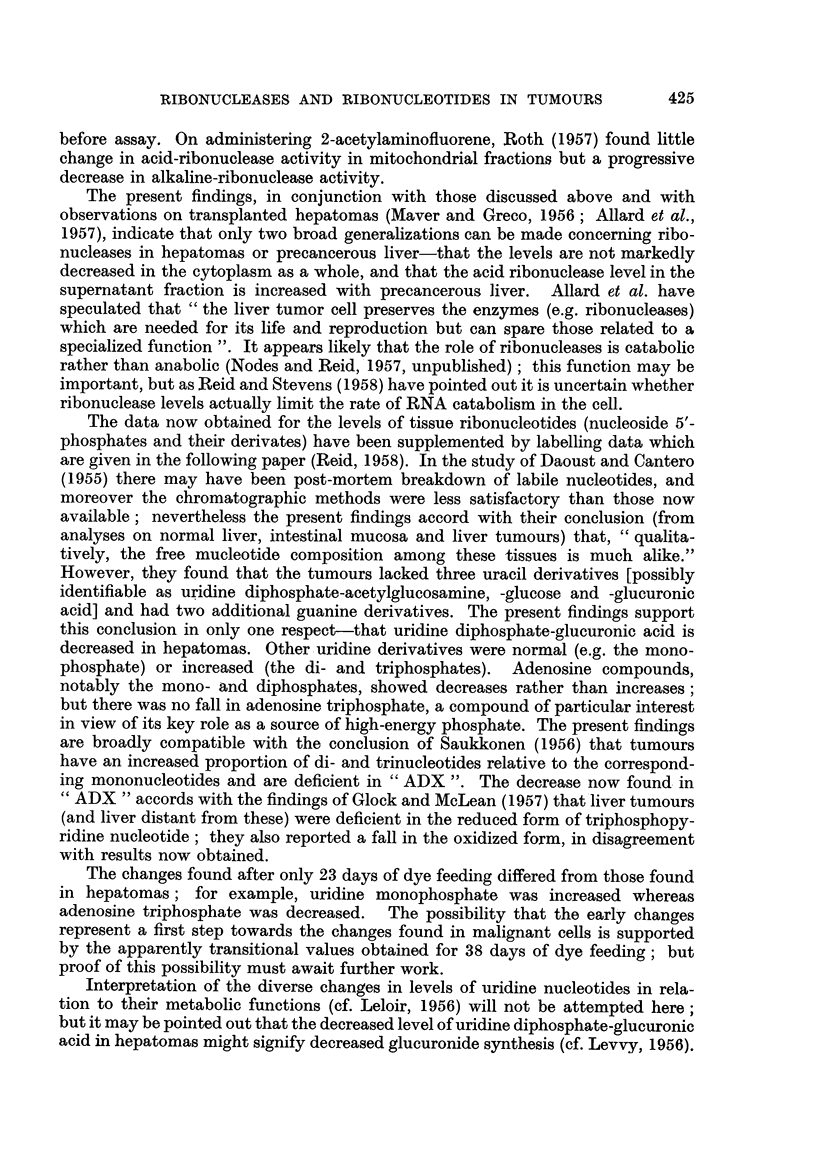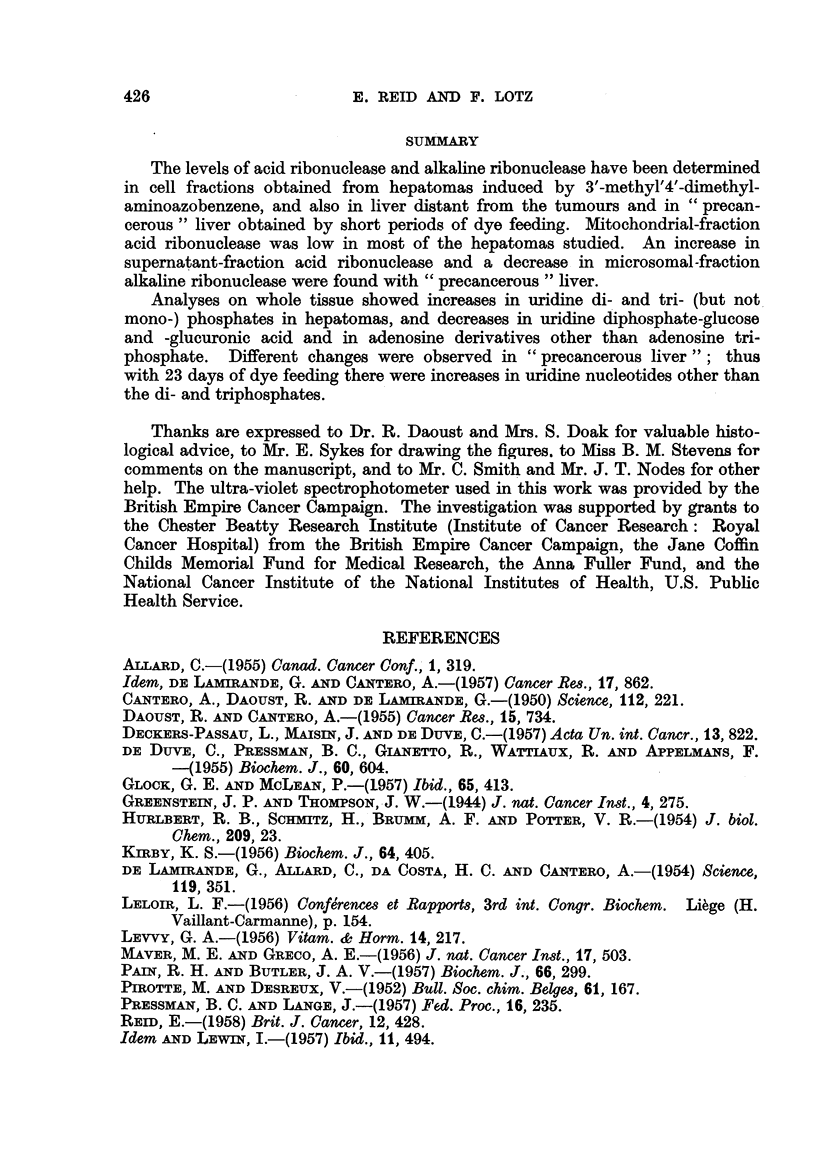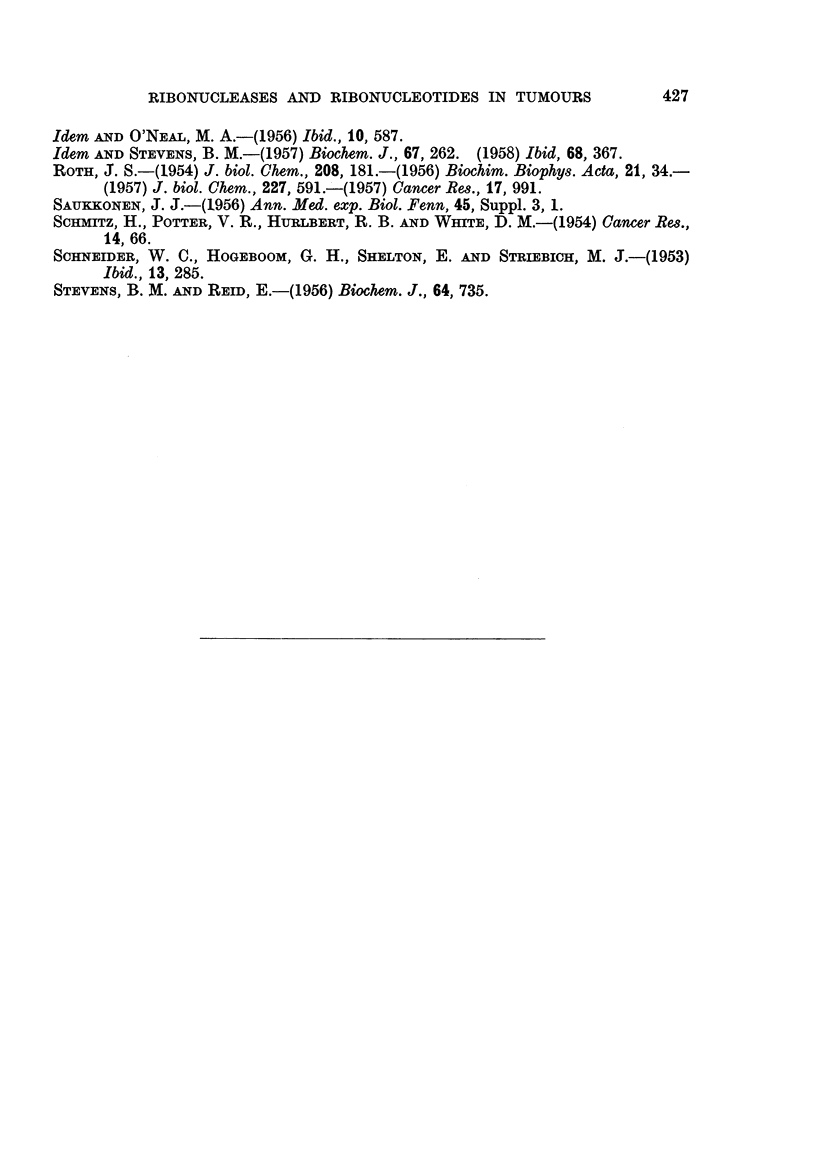# Ribonucleases and Ribonucleotides in Liver Tumours

**DOI:** 10.1038/bjc.1958.50

**Published:** 1958-09

**Authors:** E. Reid, F. Lotz


### RIBONUCLEASES AND RIBONUCLEOTIDES IN LIVER TUMOURS

E. REID AND F. LOTZ*

*From the Chester Beatty Research Institute, Institute of Cancer Research : Royal Cancer Hospital, London, S.W.3*

Received for publication June 24, 1958

REID and LEWIN (1957), in a study of certain enzymes concerned in nucleic acid catabolism, found that nucleoside phosphorylase and xanthine oxidase were decreased in liver tumours. No study was made of ribonucleases, which according to a brief published report (Allard, 1955) may be normal or even increased during carcinogenesis. Liver cytoplasm contains at least two ribonucleases, an " acid " and an " alkaline " ribonuclease (de Lamirande, Allard, da Costa and Cantero, 1954 ; Roth, 1954). Both are largely " bound " in particles which must be disrupted before assay if full activity is to be obtained (Pirotte and Desreux, 1952 ; de Duve, Pressman, Gianetto, Wattiaux and Appelmans, 1955 ; Nodes and Reid, 1957, unpublished experiments ; Roth, 1957). The supernatant fraction remaining after centrifugation of cell particles also contains some alkaline ribonuclease which is, however, accompanied by an inhibitor (Roth, 1956). Taking such difficulties into account, an independent investigation has now been made of ribonuclease levels with histologically verified hepatomas induced in rats by azo-dye feeding.

The main part of this paper deals with acid-soluble ribonucleotides, as characterized in normal liver by Hurlbert, Schmitz, Brumm and Potter (1954). These ribonucleotides (nucleoside 5′-phosphates) are known to be present in liver tumours (Daoust and Cantero, 1955) and in other tumours (Schmitz, Potter, Hurlbert and White, 1954 ; Saukkonen, 1956), but no quantitative study with liver tumours has hitherto been made.

Data are presented not only for liver tumours, but also for liver tissue (hyperplastic) distant from the tumours, and for " precancerous " liver tissue from rats fed azo-dye for periods of 38 days or less. Since any abnormalities found with these tissues may well have little bearing on the development of malignancy, the results are considered only briefly.

### EXPERIMENTAL

*Animals and tissue fractions.*—As in a previous study (Reid and O'Neal, 1956), the tissue fractions were derived from male rats fed on measured amounts of a diet which, for the experimental animals, contained 3′-methyl-4-dimethylaminoazobenzene. The carcinogen was withdrawn at least 10 days before autopsy or, in the case of the " precancerous liver " obtained by 23, 28 or 38 days of dye-feeding, 2, 1 or 4 days before autopsy. (All experiments with " precancerous " liver are treated as comparable except where the results appeared to depend on the duration of dye feeding.) No food was given on the day of autopsy. The hepatomas

* Fellow of the National Cancer Institute of Canada, on leave from the Department of Cancer Research, University of Saskatchewan, Saskatchewan.

were firm nodules, any tissue of necrotic appearance in the centre of the nodule being rejected. The samples of " liver distant from tumours " were essentially free of nodules on gross inspection.

In the ribonuclease experiments, as in the experiments of Reid and O'Neal (1956) and Reid and Lewin (1957), the tissue samples were homogenized in 0·25 M sucrose solution, and the debris (containing nuclei) obtained by centrifugation for 10 minutes at 600 *g* was again homogenized and centrifuged. The combined supernatants (cytoplasm) were centrifuged for 15 minutes at 10,000 *g* to sediment a mitochondrial fraction, which was washed once without removal of the " fluffy layer ". Further centrifugation, for 90 minutes at 14,500 *g*, gave a microsomal fraction and a supernatant fraction. The various fractions were stored frozen (— 30°) until required for assay.

*Assays for ribonucleases.*—The methods, and the choice of substrates, for assay of ribonucleases were based on the experiments of Stevens and Reid (1956) and of Nodes and Reid (1957, unpublished experiments) (cf. de Duve *et al.*, 1955); all assays were carried out in triplicate. To ensure release of " bound " activity, the tissue fractions were diluted with water and then frozen and thawed eight times. Acid ribonuclease were assayed with yeast RNA (dialysed) as substrate, of which 1 mg. in 0·4 ml. of 0·1 M acetate buffer (pH 5·0) was added to 0·3 ml. of the tissue dilution. Incubation was carried out at 37° with shaking, for 31 minutes or, with the blanks, for 1 minute. The reaction was stopped by addition of 0·7 ml. of cold 10 per cent perchloric acid solution containing 0·25 per cent uranyl acetate. After centrifuging, 0·5 ml. of the supernatant was diluted to 6 ml. for measurement of the extinction at 260 m$\mu$ (1 cm. light path). The factor 0·066 was used to convert the net increase in extinction to the activity of the original tissue sample in terms of $\mu$moles of mononucleotide formed per minute (de Duve *et al.*, 1955). The reaction was linear with respect to tissue concentration up to an extinction difference (experimental less blank) of 0·8.

In assays for alkaline ribonuclease, the diluted tissue fractions were adjusted to pH 3·5 with N-HCl, and heated for 10 minutes at 65° so as to destroy the inhibitor (Nodes and Reid, 1957, unpublished; cf. Roth, 1956) together with acid ribonuclease. The neutralized fractions were then subjected to 8 freeze-thaws as in the acid ribonuclease assays. The substrate was rat-liver RNA prepared by the method of Pain and Butler (1957) or, in a few experiments, by the method of Kirby (1956); 1·5 mg. in 0·2 ml. of 0·1 M histidine buffer pH 8·0) was added to 0·1 ml. of the tissue dilution. Incubation was carried out as for acid ribonuclease, the reaction being stopped by addition of 0·3 ml. of the perchloric acid reagent. After diluting 0·25 ml. to 4 ml., the extinction was measured at 260 m$\mu$, and the factor 0·038 used to convert the extinction increase to activity. Since the reaction was linear with respect to tissue concentration only up to an extinction difference of 0·10, any assay in which the difference exceeded this value had to be repeated with a lower tissue concentration.

*Ribonucleotides.*—The procedures were those described by Hurlbert *et al.* (1954) and by Reid and Stevens (1957), and were followed scrupulously so as to minimize breakdown of the more labile ribonucleotides. In summary, the rats were killed by decapitation, and the tissue samples were frozen in liquid nitrogen within one minute and ground to a powder in the frozen state. After extraction in the cold with perchloric acid solution, and after removal of the $KClO_4$ precipitate obtained on neutralization of the extract with KOH, gradient elution chromatography was carried out with Dowex 1 resin (formate form). Formic acid was used initially, followed by formic acid-ammonium formate mixtures ; the effluent was collected in 5 ml. portions (about 300 in each run). Certain peaks which were known to be heterogeneous were rechromatographed using ammonium formate at pH 5, after removal of the formic acid by freeze-drying and of ammonium formate by sublimation below 40° (infra-red lamp) under high vacuum (diffusion pump).

### RESULTS

The values obtained for ribonuclease activities and for ribonucleotide levels in control rats showed fair reproducibility ; however, the effect of any variations in environment or technique have been minimized by comparing each experimental rat with a control studied simultaneously, as in previous investigations (Stevens and Reid, 1956 ; Reid and O'Neal, 1956 ; Reid and Stevens, 1957 ; Reid and Lewin, 1957). With alkaline ribonuclease, the few values obtained using as substrate RNA prepared by the method of Kirby (1956) have been corrected to be comparable with those obtained using RNA prepared according to Pain and Butler (1957).

TABLE I.—*Ribonuclease Levels in Tissue Fractions*

The values are calculated as $\mu$moles mononucleotide liberated per min. per g. of tissue. The standard error is given after each mean difference ; the number of degrees of freedom and the probability that the difference could be due to chance (if < 10 per cent), are given parenthetically.

| | Prolonged azo-dye feeding | | | Brief azo-dye feeding | |
|---|---|---|---|---|---|
| | | Experimental rats : difference from corresponding controls | | | |
| Fraction | Control rats, mean value | Hepatoma | Liver distant from tumours | Control rats, mean value | Experimental rats (i.e. "pre-cancerous" liver) : difference from corresponding controls |
| | | *Acid Ribonuclease** | | | |
| Mitochondrial | 0·81 | −0·18 | +0·03 | 0·72 | −0·01 |
| | | +0·078 | ±0·102 (4) | | ±0·07 (3) |
| | | (6 ; P< 10%) | | | |
| Microsomal | 0·27 | −0·05 | +0·07 | 0·17 | +0·01 |
| | | ±0·042 (4) | +0·063 (3) | | ±0·032 (3) |
| Supernatant | 0·34 | +0·11 | +0·17 | 0·30 | +0·11 |
| | | ±0·120 (6) | ±0·081 (3) | | ±0·041 (5) |
| | | | | | (5 ; P< 5%) |
| | | *Alkaline Ribonuclease** | | | |
| Mitochondrial | 0·27 | −0·05 | −0·01 | 0·32 | −0·04 |
| | | ±0·066 (3) | ±0·023 (3) | | ±0·031 (6) |
| Microsomal | 0·14 | −0·02 | +0·06 | 0·12 | −0·04 |
| | | ±0·037 (4) | ±0·045 (2) | | ±0·012 |
| | | | | | (6 ; P< 2·5%) |
| Supernatant | 0·58 | +0·09 | +0·18 | 0·27 | −0·02 |
| | | ±0·093 (5) | ±0·130 (2) | | ±0·087 (5) |

* Nuclear fractions were not studied, since unbroken cells were present as well as nuclei. In other experiments in the Laboratory (Stevens and Reid, 1956 ; Nodes and Reid, 1957, unpublished), assays on unfractionated cytoplasm have shown satisfactory recoveries of the cytoplasmic activity in the mitochondrial, microsomal and supernatant fractions (*ca.* 93 per cent recovery of acid ribonuclease, 90 per cent recovery of alkaline ribonuclease).

*Ribonucleases.*—As is evident from Table I, hepatomas showed no consistent changes except possibly a decrease (*ca.* 20 per cent; $P < 10$ per cent) in mitochondrial-fraction acid ribonuclease activity. The values for hepatomas were particularly variable, for both enzymes, with supernatant fractions; the higher values were not peculiar to one of the two main histological types encountered (adenocarcinoma; trabecular hepatoma). With " precancerous " liver there was an increase (*ca.* 35 per cent) in the acid-ribonuclease activity of supernatant fractions, and a decrease (*ca.* 35 per cent) in the alkaline-ribonuclease activity of microsomal fractions.

*Acid-soluble ribonucleotides.*—Fig. 1 shows uridine nucleotide levels expressed as differences from the levels in controls studied simultaneously. Taking into account that the values for the controls in these and other experiments did not

FIG. 1.—Changes in acid-soluble uridine nucleotide levels (density units per g. of tissue; if a nucleotide peak when dissolved in only 1 ml. had $E_{260} = 1 \cdot 0$, 1 density unit would be present), in comparison with controls studied simultaneously.

In Fig. 1 and Fig. 2, $P_{23}$ and $P_{38}$ denote precancerous liver obtained by 23 and 38 days of dye feeding respectively (one experiment each), H denotes hepatomas (2 experiments, the individual values being shown by dots), and D denotes liver distant from tumours. Each experiment was performed with tissue samples derived from 2 rats. The standard errors, given to indicate the variability encountered in normal rats not given azo-dye, are based on the results of 6 control experiments (including experiments from other investigations in this Laboratory).

The values for uridine mono- and triphosphates were obtained by re-chromatography of heterogenous peaks, the former of which also contained inosine monophosphate. The values for uridine diphosphate and its glucuronic acid derivative were obtained by re-chromatography of a peak which also contained adenosine triphosphate.

vary widely (as judged by the standard errors in Fig. 1), it appears that with hepatomas there were increases of the order of 50 per cent (although small in absolute magnitude) in uridine di- and tri-phosphates, with no change in uridine monophosphate. On the other hand, there were decreases in uridine diphosphate-glucose and uridine diphosphate-glucuronic acid. There appeared to be similar changes in liver distant from tumours, except that uridine diphosphate-glucuronic acid was increased rather than decreased.

FIG. 2.—Changes in levels (density units per g. of tissue) of acid-soluble nucleotides other than uridine nucleotides, in comparison with controls studied simultaneously.

The values for adenosine triphosphate and inosine monophosphate were obtained by re-chromatography, as indicated in the caption to Fig. 1.

With 23 days of dye feeding there were striking increases (of the order of 100 per cent) in the levels of uridine nucleotides other than the di- and tri-phosphates ; after 38 days of dye feeding no such increases were observed except with uridine monophosphate.

Fig. 2 shows the results for adenosine nucleotides and for derivatives of these, namely triphosphopyridine nucleotide (not consistently separable from guanosine monophosphate), inosine monophosphate, and " ADX ", the latter including decomposition products of reduced triphosphopyridine nucleotide (Pressman and Lange, 1957). It is evident that hepatomas have decreased amounts of " ADX ", adenosine mono- and di-phosphates and inosine monophosphate, but not of adenosine triphosphate. The only abnormality found in liver distant from tumours was a high level of adenosine monophosphate.

With 23 days of dye feeding there were decreases in " ADX " and adenosine triphosphate, but an increase in adenosine monophosphate. With 38 days, decreases were found in adenosine mono- and diphosphates (as with hepatomas) as well as in adenosine triphosphate.

### DISCUSSION

As the present experiments were being completed, there appeared the full report (Allard, de Lamirande and Cantero, 1957) of the experiments mentioned by Allard (1955). On re-calculating their results as activity per gramme of original tissue, to be comparable with those now presented, it appears that administration of 4-dimethylaminoazobenzene led to increases (more marked in tumours than in precancerous liver) in the activities of both ribonucleases in the supernatant fraction, whereas the activities of the mitochondrial and microsomal fractions decreased. The only consistent changes now found were an increase in supernatant-fraction acid ribonuclease in precancerous liver, a decrease in microsomal-fraction alkaline ribonuclease in precancerous liver, and possibly a decrease in mitochondrial-fraction acid ribonuclease in hepatomas.

The absence of a consistent rise in supernatant-fraction alkaline ribonuclease in hepatomas apparently conflicts with the results of Allard *et al.* (1957), but may be a reflection of the high variability now encountered, reminiscent of that encountered by these authors with transplanted as distinct from primary hepatomas. It should be noted that a high (although variable) proportion of the cytoplasmic alkaline-ribonuclease activity was now found in the supernatant fraction, whereas less than one-fifth was found by Allard *et al.*, perhaps because the effect of the endogenous inhibitor persisted in the assay. Differences in centrifugation techniques, as well as in other conditions, render comparisons difficult between the two laboratories, although with the acid enzyme there is in fact good agreement with regard to the distribution in the cytoplasm of control rats. In the actual assays as now performed, not only the inhibitor but also the acid enzyme was destroyed before assaying the alkaline enzyme ; moreover the contribution of the alkaline enzyme to the observed acid ribonuclease activity was probably less than in the assays of Allard *et al.*, because of the lower pH now used (5·0 as compared with 5·8). At least it can be concluded that the variability now encountered is not due to differences in food intake, which was kept the same for control rats as for experimental rats (all rats being fasted overnight before autopsy).

Deckers-Passau, Maisin and de Duve (1957) performed careful studies on acid ribonuclease levels (per gramme of liver) in rats fed 4-dimethylaminoazobenzene. Both ' total ' activity (in the whole cytoplasm) and more especially the ' free ' activity (in the supernatant fraction) showed increases, especially at an early stage of dye-feeding and in the tumours. These changes are considered to reflect changes in the enzyme-containing particles (' lysosomes ').

In other reports published since the distinction between the two ribonucleases was recognized (cf. Greenstein and Thompson, 1944 ; Cantero, Daoust and de Lamirande, 1950), supernatant fractions were not actually assayed; however, Schneider, Hogeboom, Shelton and Striebich (1953) found no change in the acid-ribonuclease activity of mitochondrial fractions but a rise in that of whole homogenates, on feeding 3′-methyl-4-dimethylaminoazobenzene (as used in the present experiments) for 28 days ; it is not clear if all " bound " activity was released before assay. On administering 2-acetylaminofluorene, Roth (1957) found little change in acid-ribonuclease activity in mitochondrial fractions but a progressive decrease in alkaline-ribonuclease activity.

The present findings, in conjunction with those discussed above and with observations on transplanted hepatomas (Maver and Greco, 1956; Allard *et al.*, 1957), indicate that only two broad generalizations can be made concerning ribonucleases in hepatomas or precancerous liver—that the levels are not markedly decreased in the cytoplasm as a whole, and that the acid ribonuclease level in the supernatant fraction is increased with precancerous liver. Allard *et al.* have speculated that " the liver tumor cell preserves the enzymes (e.g. ribonucleases) which are needed for its life and reproduction but can spare those related to a specialized function ". It appears likely that the role of ribonucleases is catabolic rather than anabolic (Nodes and Reid, 1957, unpublished); this function may be important, but as Reid and Stevens (1958) have pointed out it is uncertain whether ribonuclease levels actually limit the rate of RNA catabolism in the cell.

The data now obtained for the levels of tissue ribonucleotides (nucleoside 5'-phosphates and their derivates) have been supplemented by labelling data which are given in the following paper (Reid, 1958). In the study of Daoust and Cantero (1955) there may have been post-mortem breakdown of labile nucleotides, and moreover the chromatographic methods were less satisfactory than those now available; nevertheless the present findings accord with their conclusion (from analyses on normal liver, intestinal mucosa and liver tumours) that, " qualitatively, the free mucleotide composition among these tissues is much alike." However, they found that the tumours lacked three uracil derivatives [possibly identifiable as uridine diphosphate-acetylglucosamine, -glucose and -glucuronic acid] and had two additional guanine derivatives. The present findings support this conclusion in only one respect—that uridine diphosphate-glucuronic acid is decreased in hepatomas. Other uridine derivatives were normal (e.g. the monophosphate) or increased (the di- and triphosphates). Adenosine compounds, notably the mono- and diphosphates, showed decreases rather than increases; but there was no fall in adenosine triphosphate, a compound of particular interest in view of its key role as a source of high-energy phosphate. The present findings are broadly compatible with the conclusion of Saukkonen (1956) that tumours have an increased proportion of di- and trinucleotides relative to the corresponding mononucleotides and are deficient in " ADX ". The decrease now found in " ADX " accords with the findings of Glock and McLean (1957) that liver tumours (and liver distant from these) were deficient in the reduced form of triphosphopyridine nucleotide; they also reported a fall in the oxidized form, in disagreement with results now obtained.

The changes found after only 23 days of dye feeding differed from those found in hepatomas; for example, uridine monophosphate was increased whereas adenosine triphosphate was decreased. The possibility that the early changes represent a first step towards the changes found in malignant cells is supported by the apparently transitional values obtained for 38 days of dye feeding; but proof of this possibility must await further work.

Interpretation of the diverse changes in levels of uridine nucleotides in relation to their metabolic functions (cf. Leloir, 1956) will not be attempted here; but it may be pointed out that the decreased level of uridine diphosphate-glucuronic acid in hepatomas might signify decreased glucuronide synthesis (cf. Levvy, 1956).

SUMMARY

The levels of acid ribonuclease and alkaline ribonuclease have been determined in cell fractions obtained from hepatomas induced by 3′-methyl′4′-dimethylaminoazobenzene, and also in liver distant from the tumours and in " precancerous " liver obtained by short periods of dye feeding. Mitochondrial-fraction acid ribonuclease was low in most of the hepatomas studied. An increase in supernatant-fraction acid ribonuclease and a decrease in microsomal-fraction alkaline ribonuclease were found with " precancerous " liver.

Analyses on whole tissue showed increases in uridine di- and tri- (but not mono-) phosphates in hepatomas, and decreases in uridine diphosphate-glucose and -glucuronic acid and in adenosine derivatives other than adenosine triphosphate. Different changes were observed in " precancerous liver " ; thus with 23 days of dye feeding there were increases in uridine nucleotides other than the di- and triphosphates.

Thanks are expressed to Dr. R. Daoust and Mrs. S. Doak for valuable histological advice, to Mr. E. Sykes for drawing the figures, to Miss B. M. Stevens for comments on the manuscript, and to Mr. C. Smith and Mr. J. T. Nodes for other help. The ultra-violet spectrophotometer used in this work was provided by the British Empire Cancer Campaign. The investigation was supported by grants to the Chester Beatty Research Institute (Institute of Cancer Research : Royal Cancer Hospital) from the British Empire Cancer Campaign, the Jane Coffin Childs Memorial Fund for Medical Research, the Anna Fuller Fund, and the National Cancer Institute of the National Institutes of Health, U.S. Public Health Service.

### REFERENCES

Allard, C.—(1955) *Canad. Cancer Conf.*, **1**, 319.
*Idem*, de Lamirande, G. and Cantero, A.—(1957) *Cancer Res.*, **17**, 862.
Cantero, A., Daoust, R. and de Lamirande, G.—(1950) *Science*, **112**, 221.
Daoust, R. and Cantero, A.—(1955) *Cancer Res.*, **15**, 734.
Deckers-Passau, L., Maisin, J. and de Duve, C.—(1957) *Acta Un. int. Cancr.*, **13**, 822.
de Duve, C., Pressman, B. C., Gianetto, R., Wattiaux, R. and Appelmans, F. —(1955) *Biochem. J.*, **60**, 604.
Glock, G. E. and McLean, P.—(1957) *Ibid.*, **65**, 413.
Greenstein, J. P. and Thompson, J. W.—(1944) *J. nat. Cancer Inst.*, **4**, 275.
Hurlbert, R. B., Schmitz, H., Brumm, A. F. and Potter, V. R.—(1954) *J. biol. Chem.*, **209**, 23.
Kirby, K. S.—(1956) *Biochem. J.*, **64**, 405.
de Lamirande, G., Allard, C., da Costa, H. C. and Cantero, A.—(1954) *Science*, **119**, 351.
Leloir, L. F.—(1956) *Conférences et Rapports, 3rd int. Congr. Biochem.* Liège (H. Vaillant-Carmanne), p. 154.
Levvy, G. A.—(1956) *Vitam. & Horm.* **14**, 217.
Maver, M. E. and Greco, A. E.—(1956) *J. nat. Cancer Inst.*, **17**, 503.
Pain, R. H. and Butler, J. A. V.—(1957) *Biochem. J.*, **66**, 299.
Pirotte, M. and Desreux, V.—(1952) *Bull. Soc. chim. Belges*, **61**, 167.
Pressman, B. C. and Lange, J.—(1957) *Fed. Proc.*, **16**, 235.
Reid, E.—(1958) *Brit. J. Cancer*, 12, 428.
*Idem* and Lewin, I.—(1957) *Ibid.*, **11**, 494.

*Idem* AND O'NEAL, M. A.—(1956) *Ibid.*, **10**, 587.
*Idem* AND STEVENS, B. M.—(1957) *Biochem. J.*, **67**, 262. (1958) *Ibid*, **68**, 367.
ROTH, J. S.—(1954) *J. biol. Chem.*, **208**, 181.—(1956) *Biochim. Biophys. Acta*, **21**, 34.—(1957) *J. biol. Chem.*, **227**, 591.—(1957) *Cancer Res.*, **17**, 991.
SAUKKONEN, J. J.—(1956) *Ann. Med. exp. Biol. Fenn*, **45**, Suppl. 3, 1.
SCHMITZ, H., POTTER, V. R., HURLBERT, R. B. AND WHITE, D. M.—(1954) *Cancer Res.*, **14**, 66.
SCHNEIDER, W. C., HOGEBOOM, G. H., SHELTON, E. AND STRIEBICH, M. J.—(1953) *Ibid.*, **13**, 285.
STEVENS, B. M. AND REID, E.—(1956) *Biochem. J.*, **64**, 735.